# Hand bone loss as an outcome measure in established rheumatoid arthritis: 2-year observational study comparing cortical and total bone loss

**DOI:** 10.1186/ar2280

**Published:** 2007-08-17

**Authors:** Mari Hoff, Glenn Haugeberg, Tore K Kvien

**Affiliations:** 1 Norwegian University of Science and Technology, MTFS, Department of Neuroscience, Division of Rheumatology, NO-7489 Trondheim, Norway; 2 Department of Rheumatology, St Olav's Hospital, University Hospital of Trondheim, Olav Kyrres gt 17, N-7006 Trondheim, Norway; 3 Department of Rheumatology, Sørlandet Hospital, Service box 416, N-4604 Kristiansand S., Norway; 4 Department of Rheumatology, Diakonhjemmet Hospital, PB 23 Vinderen, N-0319 Oslo, Norway; 5 Faculty of Medicine, University of Oslo, PB 1072 Blindern, N-0316 Oslo, Norway

## Abstract

The aim of this 2-year longitudinal observational study was to explore hand bone loss as a disease outcome measure in established rheumatoid arthritis (RA).

A cohort of 215 patients with RA (170 women and 45 men, aged 20–70 years) were recruited from the Oslo RA registry and studied for changes in hand bone mass during a 2-year follow-up. Digital X-ray radiogrammetry (DXR) was used to measure cortical hand bone mineral density (BMD) and metacarpal cortical index, whereas dual-energy X-ray absorptiometry (DXA) was used to assess whole hand BMD, which measures total cortical and trabecular bone. DXA-BMD total hip and spine and informative data for disease and therapy were also collected.

Hand bone loss could be revealed over a 2-year follow-up measured by DXR-BMD (-0.90%, *P *< 0.01), but not by DXA-BMD (0.00%, *P *= 0.87). DXA-BMD hand bone loss was only observed in patients with disease duration ≤3 years and not in patients with longer disease duration (-0.96% versus 0.24%, *P *< 0.01), whereas loss of DXR-BMD was independent of disease duration. Disease activity (measured by the disease activity score including 28 joints) independently predicted loss of DXR-BMD but not changes in the DXA-BMD hand in the multivariate analysis. The change in DXR metacarpal cortical index was highly correlated to DXR-BMD (*r *= 0.94, *P *< 0.001).

These data suggest that DXR-BMD may be a more appropriate technique to identify RA-related bone involvement in hands compared with DXA-BMD measurement, but further studies are needed to explore this hypothesis.

## Introduction

Periarticular bone loss and erosions on radiographs are characteristic features of bone damage in rheumatoid arthritis (RA) [1], and both features are caused by joint inflammation [2]. Substantial data suggest a common cellular pathway for both periarticular bone loss and erosions involving the osteoclast cell [3,4]. In active RA there is an excess production of proinflammatory cytokines (for example, IL-1 and TNFα), which stimulates receptor activator of nuclear factor kB ligand (RANKL) to activate the osteoclast cell [3-5].

Because periarticular bone loss is an early finding and may also precede erosions on radiographs [6], quantitative hand bone measurements that capture periarticular osteoporosis have been proposed as outcome measures in early RA [7,8]. Inflammation of the joints, however, is not restricted to the early phase of the RA disease, but may be present during the entire disease course [9]. Hand bone loss could therefore potentially be an outcome measure in RA patients with prolonged disease.

Several devices for quantitative bone measurements have been developed [10] – for example, quantitative computer tomography, measuring cortical and trabecular bone separately; quantitative ultrasound, providing measures that may reflect bone quality; dual-energy X-ray absorptiometry (DXA), which measures total cortical and trabecular bone; and digital X-ray radiogrammetry (DXR), which measures cortical bone only. DXA is considered the gold standard among bone measurement devices for assessment of bone density at the hip and the spine. DXA has not, however, been shown to be superior to other bone measure devices, such as DXR, in the hand [11]. DXR, which is a further development and digitalized version of the conventional radiogrammetry [12], is a new promising method for assessment of cortical hand bone loss [13].

The understanding of hand bone loss as an outcome measure in RA is mainly limited both due to lack of data from longitudinal studies and due to the small number of patients included in previous studies. Only a few studies have examined associations between disease factors and hand bone loss in RA, and most of them have focused on patients with early disease [6-8,11,14,15]. Data from two longitudinal studies by Deodhar and colleagues suggest that whole hand DXA bone mineral density (BMD) loss only takes place in the first 2–3 years of the RA disease process, which may limit the use of hand DXA-BMD as an outcome measure in prolonged disease [7,15]. Only a few studies have compared hand DXA-BMD with hand cortical bone DXR-BMD in RA [11,16].

The aim of the present study was to explore hand bone loss as a disease outcome measure in established RA assessed by DXR and by DXA and to compare the two methods.

## Materials and methods

### Patients

The 215 RA patients (45 males and 170 females) included in the present study were recruited from a longitudinal cohort of 366 RA patients (aged 20–70 years) [17], all patients fulfilling the American College of Rheumatology (ACR) criteria and enrolled in the Oslo RA register [18]. Two-year changes in generalized bone loss at the hip and the spine from this original cohort have previously been described in detail [17]. In the present study, only patients with hand radiographs and DXA-BMD measurement of the hand at baseline and 2-year follow-up were included; 151 patients missed at least one BMD measurement and were excluded. There were no other exclusion criteria.

### Demographic and clinical variables

The demographic and clinical characteristics of the patients (Table 1) were recorded by a combination of self-reported questionnaires, interview and clinical investigation, as previously reported [17]. In short, the clinical examination included 28-swollen and tender joint counts as well as routine laboratory tests. The disease activity score including 28 joints (DAS28) was computed based on the erythrocyte sedimentation rate [19]. Patients with a titer ≥64 of the Waaler–Rose reaction were classified as rheumatoid factor-positive. The physician's global assessment of disease activity was measured on a visual analogue scale (0–100 mm). Use of antiresorptive osteoporotic treatment (AOT) with bisphosphonates or hormone replacement therapy, prednisolone and disease-modifying antirheumatic drugs (DMARD) was recorded. Physical disability was measured by the Modified Health Assessment Questionnaire (MHAQ) (eight items; range of scores 1–4) [20].

**Table 1:** Patient characteristics at baseline and at 2-year follow-up

Variable	*n*	Baseline	At 2-year follow-up
Demographic			
Age (years)	215	57.4 (49.1–64.7)	
Female	215	170 (79.0%)	
Menopause	170	111 (65.3%)	
Body mass index (kg/m^2^)	215	23.9 (21.3–26.2)	24.0 (21.5–26.2)
Smoker	210	65 (31.0%)	67 (31.9%)
Disease			
Disease duration (years)	215	9 (4–16)	
Age at disease onset (years)	215	45.0 (33.0–53.0)	
Rheumatoid factor-positive	202	97 (48.0%)	
Physician's global assessment score (visual analogue scale, 0–100 mm)	203	19.0 (8.0–39.8)	17.6 (8.5–30.0)
Modified Health Assessment Questionnaire (range 1–4)	214	1.50 (1.13–1.75)	1.50 (1.13–1.87)
Erythrocyte sedimentation rate (mm/hour)	210	16 (9–27)	14 (8–27)
Disease activity score including 28 joints	202	4.04 (3.17–4.96)	4.26 (3.36–5.06)
Medication			
Ever user of disease-modifying antirheumatic drugs	213	177 (83.1%)	177 (83.1%)
Corticosteroids	208	79 (37.9%)	85 (40.9%)
User of corticosteroids in the 2-year period	208		93 (44.7%)
Antiresorptive osteoporosis treatment	209	47 (22.5%)	68 (32.5%)
Ever user of antiresorptive osteoporosis treatment	209		92 (44.0%)
Calcium and/or vitamin D	210	113 (53.8%)	155 (73.8%)

Data presented as the median (interquartile range) or as the absolute value (%).

### Bone mineral density measurements

The DXR-BMD and the DXR metacarpal cortical index (MCI) was measured by the Pronosco X-posure system™ (version 2.0; SECTRA, Linköping, Sweden) [13], a computer version of the traditional technique of radiogrammetry [12]. The computer automatically recognizes, on standard radiographs, regions of interest around the narrowest part of the second, third and fourth metacarpal bones of the hand. In each region, the cortical thickness, bone width and porosity is measured 118 times per centimeter. The final BMD estimate is defined as: DXR-BMD = *c *× VPA_comb _× (1 – *p*), where *c *is a constant (determined such that DXR-BMD on average is equal to the mid-distal forearm region of the Hologic QDR-2000 device (Hologic Inc., Bedford, MA, USA)), VPA is the volume per area and *p *is the porosity. The DXR method has previously been described in detail [13,21]. The MCI is defined as the combined cortical thickness divided by the outer cortical diameter and is a relative measure independent of bone size or bone length [22,23]. The DXR method has improved the precision of MCI for diagnosing cortical bone loss [12,23]. All radiographs of the hand were acquired by a Siemens Multix Polymat equipment (Siemens AG, Erlangen, Germany) (AGFA Curix film; film focus distance, 1 m; X-ray tube voltage, 55 kV; exposure dose, 6 mAs).

Standardized BMD measurements for the left and right hands and the total hip and spine (L2–L4) were performed using the same DXA equipment (Lunar Expert; Lunar Corporation, Madison, WI, USA) both at baseline and follow-up. All procedures were in accordance with the manufacturer's standardized procedures for hand BMD measurements.

For the DXR-BMD most previous studies have used the nondominant hand [11,14], while for DXA measures there is no consistency and both hands [8], the right hand [15,24] and the nondominant hand [11] have all been used. To avoid bias regarding dominant and nondominant hands and to achieve better precision, we used the mean of both hands. Only patients who had complete measurements for both DXA-BMD and DXR-BMD in both hands were therefore included.

### Precision of bone mineral density measurements

Short-time precision was calculated from the material of 28 healthy individuals who underwent duplicate hand BMD measurements and duplicate hand radiographs of both hands with repositioning of the hand between each assessment. Short-time precision based on the duplicate measurements, expressed as the percentage coefficient of variation, was 0.28% for the DXR-BMD hand and was 0.76% for the DXA-BMD hand. Long-time precision for DXR-BMD based on daily measurement of one hand radiograph was 0.25%, and long-time precision for the DXA-BMD hand based on daily measurements of the aluminum spine phantom supported by the Lunar Expert (Lunar Corporation) was 0.80%

### Ethics and legal aspects

The study was approved by the regional committee for ethics and medical research.

The Norwegian Data Inspectorate approved the registry of RA patients in Oslo.

### Statistical analysis

The statistical analyses were carried out using the SPSS program, version 13 (SPSS Inc., Chicago, IL, USA). Nonparametric tests were used for comparisons between groups (Mann–Whitney and Kruskal–Wallis tests) and within groups (Wilcoxon test) because of a skewed distribution of data. Results are presented as the median and interquartile range (25th–75th percentiles). Bivariate correlations were tested using Spearman's correlation.

Bone loss over time was expressed as a negative value. Changes of BMD measurements were compared across groups according to the disease duration (cut-off 3 years), baseline DAS28 (<3.2, low disease activity; 3.2–5.1, moderate disease activity; >5.1, high disease activity) and baseline MHAQ score (<1.50, 1.50–1 99, ≥2). The 3-year cut-off value for disease duration was chosen for pragmatic reasons due to a low number of included patients with short disease duration and reports in the literature suggesting hand bone loss only takes place in the first 3 years of disease duration [7].

The predictive values of disease duration, DAS28 and MHAQ score were also tested in a multiple linear regression model, with the change of hand BMD as the dependent variable and with adjustments for age, gender, rheumatoid factor and use of medication (AOT, prednisolone and DMARD). Enter and stepwise procedures were used. According to inspection of Q–Q plots, the distribution of residuals showed acceptable fit to the normal distribution regarding hand DXR-BMD, whereas one outlier was identified in the analysis with hand DXA-BMD as the dependent variable. This analysis was therefore performed both with and without the outlier.

Two tailed *P *values of 0.05 or less were considered statistically significant.

## Results

Patient characteristics at baseline and at follow-up are presented in Table 1. The 215 examined patients in this study had shorter disease duration (9 years versus 15 years, *P *< 0.01), lower disease activity measured by the DAS28 (4.00 versus 4.62, *P *< 0.01), lower global assessment (19 versus 30, *P *< 0.01) and used less prednisolone (37% versus 54%, *P *< 0.01) compared with those who were not included (*n *= 151) from the original cohort (*n *= 366). The two groups were similar regarding age, gender, body mass index, smoking habits, rheumatoid factor, age of disease onset, erythrocyte sedimentation rate, menopause in women and use of DMARD and AOT.

### Change in bone mineral density

**Figure 1:**
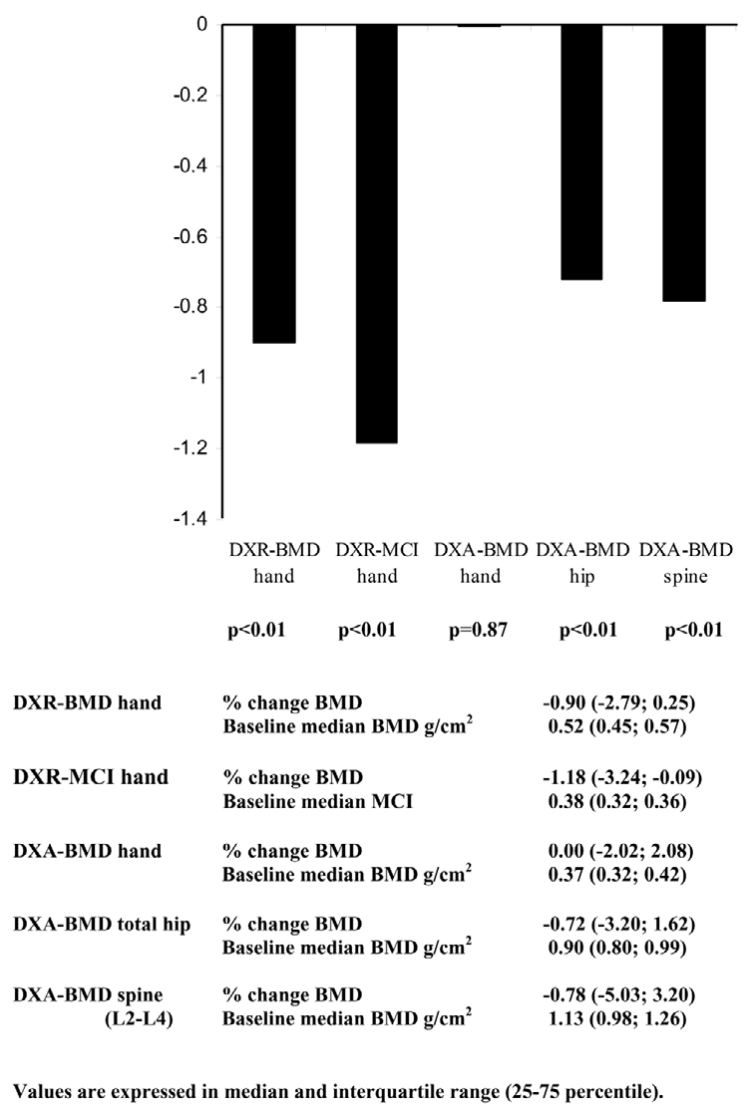
Bone loss in 215 rheumatoid arthritis patients followed for 2 years. Bone loss assessed by digital X-ray radiogrammetry (DXR) bone mineral density (BMD) and metacarpal cortical index (MCI) of the hand, and by dual-energy X-ray absorptiometry (DXA) BMD of the hand, total hip and spine (L2–L4).

In the entire group, a significant loss in hand BMD was seen at 2 years as measured by DXR-BMD (-0.90%) and DXR-MCI (-1.18%), but not as measured in the DXA-BMD hand (0.00%) (Figure 1). A significant bone loss was also observed for the DXA-BMD in the total hip (-0.72%) and in the spine L2–L4 (-0.78%) (Figure 1).

The correlation (*r *value) between the DXR-BMD hand and the DXA-BMD hand was 0.88 (*P *< 0.001) for baseline values and was 0.35 (*P *< 0.001) for 2-year BMD changes. Correlations between the change in the DXA hand and in the DXA total hip and spine were 0.35 (*P *< 0.001) and 0.18 (*P *= 0.01), whereas correlations between the change in the DXR hand and DXA total hip and spine were 0.23 (*P *= 0.001) and 0.10 (*P *= 0.16), respectively. The DXR-MCI was highly correlated with the DXR-BMD both at baseline (*r *= 0.86, *P *< 0.001) and as the percentage change over 2 years (*r *= 0.94, *P *< 0.001).

### Association between disease duration and bone loss

At baseline 37 patients had a disease duration of 3 years or less and 178 patients had a disease duration longer than 3 years. DXA-BMD hand bone loss was only observed in patients with disease duration less than 3 years and not in patients with longer disease duration (-0.96% versus 0.24%, *P *< 0.01) (Table 2), whereas loss of DXR-BMD (-0.46% versus -0.93%, *P *= 0.76) as well as loss of DXR-MCI (-0.89 versus -1.29, *P *= 0.66), of the DXA-BMD total hip (-0.26% versus -0.76%, *P *= 0.51) and of the DXA-BMD spine (-0.71% versus -0.82%, *P *= 0.64) occurred independent of disease duration. The changes in BMD in the subgroups (according to disease duration) were all significant except for the DXA-BMD hand patients with disease duration longer than 3 years (*P *= 0.26) and the DXA-BMD spine (*P *= 0.60) and DXA-BMD total hip patients with disease duration less than 3 years (borderline significant, *P *= 0.06).

**Table 2:** Comparison of the baseline and the change in hand bone mineral density

	Disease duration			DAS28 at baseline				MHAQ at baseline			
	
	≤3 years	>3 years	*P *value*	<3.2	3.2–5.1	>5.1	*P *value	<1.50	1.50–1.99	≥2.0	*P *value
*n*	37	178	<0.01	55	103	44	<0.01	102	78	34	<0.01
Age (years)	55.4 (43–62)	58.0 (50–65)	0.10	53.5 (39–61)	55.8 (49–64)	62.2 (57–67)	<0.01	53.6 (41–64)	58.9 (52–64)	61.2 (54–67)	<0.01
DXA-BMD (g/cm^2^)	0.39 (0.34–0.43)	0.36 (0.31–0.41)	0.04	0.40 (0.36–0.43)	0.38 (0.32–0.42)	0.33 (0.28–0.38)	<0.01	0.38 (0.33–0.43)	0.37 (0.31–0.41)	0.34 (0.30–0.39)	0.13
DXA-BMD change (%)	-0.96 (-4.4 to 1.5)	0.24 (-1.4 to 2.1)	<0.01	-0.40 (-2.4 to 1.8)	0.26 (-1.3 to 2.2)	0.04 (-3.4 to 2.2)	0.40	0.11 (-2.5 to 2.1)	0.0 (-1.2 to 2.0)	-0.12 (-4.1 to 2.2)	0.75
DXR-BMD (g/cm^2^)	0.57 (0.50–0.61)	0.51 (0.44–0.56)	<0.01	0.56 (0.50–0.61)	0.53 (0.45–0.58)	0.46 (0.38–0.52)	<0.01	0.54 (0.49–0.59)	0.49 (0.44–0.57)	0.50 (0.40–0.53)	<0.01
DXR-BMD change (%)	-0.46 (-3.6 to 0.2)	-0.93 (-2.8 to 0.3)	0.76	-0.29 (-1.6 to 0.7)	-1.13 (-3.2 to 0.1)	-1.03 (-4.3 to 0.5)	0.03	-0.80 (-2.6 to 0.1)	-0.94 (-2.8 to 0.5)	-0.81 (-3.7 to 0.5)	0.90
DXR-MCI	0.40 (0.37–0.49)	0.37 (0.31–0.45)	<0.01	0.41 (0.34–0.48)	0.39 (0.33–0.46)	0.32 (0.27–0.38)	<0.01	0.40 (0.33–0.48)	0.37 (0.31–0.43)	0.33 (0.29–0.41)	<0.01
DXR-MCI change (%)	-0.89 (-5.5 to 0.0)	-1.29 (-3.1 to -0.1)	0.66	-0.76 (-1.8 to 0.3)	-1.34 (-3.4 to -0.4)	-1.13 (-5.2 to -0.2)	0.06	-1.33 (-3.1 to -0.3)	-1.20 (-3.2 to 0.3)	-0.71 (-5.0 to 0.0)	0.74

Digital X-ray radiogrammetry (DXR) bone mineral density (BMD), DXR metacarpal cortical index (MCI) and dual-energy X-ray absorptiometry (DXA) BMD assessed for levels of disease duration, for disease activity (disease activity score including 28 joints (DAS28)) and for physical function (Modified Health Assessment Questionnaire (MHAQ)) in rheumatoid arthritis patients. Data presented as the medians (interquartile range). *P values between subgroups.

The patients with short and long disease duration were comparable with regard to demographic variables, disease activity and treatment with DMARD and corticosteroids, but AOT was used less frequently by patients with short disease duration than by patients with long disease duration (16.1% versus 35.5%, *P *= 0.04). The difference in DXA hand bone loss across patients with short and long disease duration, however, was also significant in the subgroup not using AOT (-1.41% versus 0.11%, *P *= 0.02). These findings are consistent in a linear regression model adjusted for other variables that may influence hand bone loss (Table 3). The analysis was performed both with and without the outlier, with the same results.

**Table 3:** Risk factors for hand bone loss in a multivariate linear regression model

	DXA-BMD hand percentage change		DXR-BMD hand percentage change		DXR-MCI percentage change	
	
	*B *(standard error)	*P *value	*B *(standard error)	*P *value	*B *(standard error)	*P *value
Disease activity score including 28 joints	0.09 (0.25)	0.73	-0.47 (0.16)	0.003	-0.47 (0.18)	0.009
Disease duration <3 years	-2.84 (0.88)	0.001	0.46 (0.55)	0.40	0.45 (0.63)	0.47
Baseline BMD (g/cm^2^)/MCI	-9.70 (5.01)	0.05	-3.80 (2.51)	0.13	-5.79 (2.81)	0.04
Prednisolone during 2-year follow-up (no/yes)	0.44 (0.69)	0.53	-0.03 (0.43)	0.95	-0.41 (0.49)	0.40
Ever disease-modifying antirheumatic drug user (no/yes)	-0.31 (0.90)	0.73	-0.58 (0.55)	0.30	-0.56 (0.63)	0.38
Ever antiresorptive osteoporosis treatment user (no/yes)	0.78 (0.70)	0.27	0.03 (0.42)	0.95	-0.05 (0.47)	0.91
*R*^2^	0.11		0.05		0.06	

*B *values are unstandardized coefficients. Age, gender, rheumatoid factor and the Modified Health Questionnaire were also tested, but did not influence the results. DXA, dual-energy X-ray absorptiometry; DXR, digital X-ray radiogrammetry; BMD, bone mineral density; MCI, metacarpal cortical index.

### Association between disease activity score and hand bone loss

At baseline 55 patients had low disease activity, 103 patients had moderate disease activity and 44 patients had high disease activity. Bone loss changes, as measured by DXR-BMD, differed across patients with different levels of disease activity (low, -0.29%; moderate, -1.13%; and high, -1.03%; *P *= 0.03), and were borderline significant for DXR-MCI (-0.76, -1.34 and -1.13, *P *= 0.06) (Table 2). No significant difference in DXA-measured hand BMD change was found for the low, moderate and high levels of disease activity (-0.40% versus 0.26% versus 0.04%, respectively; *P *= 0.40). Hand BMD baseline values, however, were significantly lower in the group with high disease activity in both the DXR-BMD and the DXA-BMD (Table 2).

The correlation (*r *value) between the DAS28 at baseline (continuous scale) and the hand DXR-BMD change was -0.19 (*P *= 0.01), between the DAS28 and the DXR-MCI change was -0.16 (*P *= 0.03), and between the DAS28 and the hand DXA-BMD change was 0.08 (*P *= 0.27). Patients in the group with high disease activity were significant older than the group with lowest disease activity. In a multivariate model, however, disease activity was independently associated with the percentage change in DXR-BMD (*B *= -0.47, *P *< 0.01) (Table 3) and with the DXR-MCI (*B *= -0.47, *P *< 0.01), after adjusting for other variables that could influence hand bone change as well as age.

### Association between functional disability (MHAQ score) and hand bone loss

At baseline, 102 patients had a MHAQ score less than 1.50, 78 patients a score between 1.50 and 1.99, and 34 patients had a MHAQ score of two or more. The patient with highest MHAQ score was older than patients with lower MHAQ scores. Regarding correlation between the MHAQ score at baseline and the change in hand DXR-BMD, the DXR-MCI hand and the DXA-BMD hand were nonsignificant both for continuous values (*r *= 0.00, *P *= 0.96; *r *= 0.03, *P *= 0.70; and *r *= -0.05, *P *= 0.51) and for groups (*r *= 0.02, *P *= 0.82; *r *= 0.05, *P *= 0.47; and *r *= -0.02, *P *= 0.82) for the MHAQ score ranges <1.5, 1.50–1.99 and ≥2, respectively. There were no differences in the change in hand BMD dependent on the MHAQ group either in the DXR-BMD hand, the DXR-MCI hand or the DXA-BMD hand. Baseline values, however, were significantly higher in the group with the lowest MHAQ score with regards to DXR-BMD and DXR-MCI (Table 2). No such findings were seen regarding DXA measurements.

### Associations between treatment and hand bone loss

At follow-up 33% of the patients were current users of AOT (88% used hormone replacement therapy and 12% used bisphosphonates) and 44% were ever users. A significant difference in DXA-BMD hand change was found between users and nonusers of AOT (0.44% versus 0%, *P *= 0.04). No such difference was seen for DXR-BMD (-1.01% versus -0.66%, *P *= 0.54) or DXR-MCI (-1.14 versus -1.19, *P *= 0.60) in users versus nonusers of AOT. Use of AOT, however, was not significantly associated with the change in DXA-BMD in the multivariate analyses (Table 3).

No significant difference in hand bone change was seen between ever users (83%) and never users (17%) of DMARD regarding the DXR-BMD hand (-0.90% versus -0.85%, *P *= 0.29), the DXR-MCI hand (-1.19 versus -0.78, *P *= 0.17) or the DXA-BMD hand (0.27% versus -0.34%, *P *= 0.22). During the 2-year follow-up 45% of patients had used prednisolone and 41% were current users at follow-up with a mean dose of 5.7 mg. No significant difference in change of hand BMD was observed between users and nonusers of prednisolone regarding DXR-BMD (-0.94% versus -0.66%, *P *= 0.19) or DXA-BMD (0.62% versus 0%, *P *= 0.17), but a group difference between users and nonusers was observed for DXR-MCI (-1.42 versus -0.98, *P *= 0.05). Prednisolone users, however, had a significantly higher disease activity than nonusers (data not shown) and the significant association between prednisolone and the change in DXR-MCI disappeared in the multivariate analysis (Table 3).

## Discussion

The present study had two main findings. First, total hand bone loss measured by DXA-BMD seems to occur only in the first years of RA disease, whereas DXR-BMD-measured cortical hand bone loss occurs both in early stages as well as late stages of the disease. Second, patients with high disease activity at baseline lost more DXR-BMD and DXR-MCI than patients with low disease activity. In the present study there were only marginally differences between DXR-BMD and DXR-MCI, and our main focus in the discussion will therefore be on DXR-BMD.

A discrepancy in loss of DXA-BMD hand between early disease and long-standing disease has previously been suggested based on the results of two longitudinal studies [7,15]. Hand bone loss was only observed in the first 3 years and then stabilized over the next 2 years in a longitudinal study of 29 patients with RA [7]. Degenerative bone changes and increased inflammation in the small joints of the hand in the first years of the disease has been suggested partly to explain this finding [25]. As DXA-BMD measures both trabecular and cortical bone, a third explanation could be that the rate of trabecular and cortical bone loss is different in early stages versus late stages of the disease. Even if DXR-BMD hand bone loss occurs during the whole RA disease course, the bone loss has been shown to be more rapid in early disease compared with more prolonged disease [14]. Böttcher and colleagues reported annual DXR-BMD loss in the first 6 years of the disease to be as high as 11%, with a subsequent decline to 3–4% over the next years [14].

Interestingly, changes in the DXA-BMD in the total hip and spine were independent of the disease duration. There are few studies that have compared periarticular and generalized osteoporosis among RA patients [8,26-28]. Hand bone loss in early RA has been shown to occur more rapidly than bone loss in the hip and the spine [8,28]. Radiographic joint damage has been shown to be more strongly correlated with low hand DXR-BMD than DXA-BMD at the hip and the spine [26,27]. In a randomized, placebo-controlled trial among early RA patients, use of prednisolone reduced hand bone loss [29]. These data suggest that the effect of inflammation on hand bone in RA may be greater than the effect on other bones (for example, spine and hip). The generalized bone loss may be more associated with the prolonged course of RA, including the use of corticosteroids and immobility [30].

The other main finding in the present study is that patients with high disease activity at baseline lost more DXR-BMD than patients with low disease activity. Surprisingly, this association was not found between DXA-BMD hand bone loss and baseline disease activity, and this lack of association was consistent in both patients with short and long disease durations (data not shown). Some previous studies in early RA, however, have shown that disease activity is associated with both DXA-BMD-measured generalized bone loss [31] as well as localized bone loss [8]. Gough and colleagues [31] found that early RA patients with active disease (defined as mean C-reactive protein >20 mg/l over 12 months) showed greater generalized bone loss at the hip and the spine compared with patients with lower disease activity. Haugeberg and coworkers [8] found that C-reactive protein independently predicted hand BMD loss in patients with early undifferentiated arthritis who, during a 12-month follow-up, developed RA. Explanations for contradictory findings between these two studies and our study may be differences in disease activity and disease duration in the examined study cohorts.

The association between disease activity and DXR-BMD hand bone loss in our study was shown when dichotomizing the patients into groups based on disease activity (Table 2) and in linear multivariate analyses (Table 3). These consistent associations combined with the demonstration of bone loss independent of disease duration (Table 3) suggest that DXR-BMD is a robust outcome measure in RA, reflecting the inflammatory disease process in early stages as well as late stages of the disease. Only a few previous studies have been carried out with DXR-BMD loss as the key outcome measure [11,32]. Jensen and colleagues [11] found in patients with early RA (<2 years) that DXR-BMD was more strongly associated with disease activity than hand DXA-BMD. In a cross-sectional study, Böttcher and colleagues found that DXR-BMD was negatively correlated with disease activity measured by the DAS28 [32].

In the present study the hand bone loss measured by both DXR-BMD and DXA-BMD was less than that reported by other workers. Jensen and colleagues [11] found a loss of DXR-BMD of 5% over 2 years in an early RA disease group, and Haugeberg and colleagues found that the DXA-BMD hand loss was reduced by 4.3% in early RA disease patients [8]. One explanation for the lower rate of hand bone loss in the present study may be that our cohort was obtained from an observational study of patients with different levels of disease activity and duration. The recruitment of these patients from a validated RA register is also a strength of the present study as the results provide insight into what takes place in the real world of RA patients regarding hand bone loss [18]. Another reason for the less bone loss may be that the DXA-BMD hand was assessed as a whole hand and not around selected finger joints, which according to the cross-sectional study by Alenfeld and colleagues [33] has been suggested to be the best site to capture periarticular bone loss in RA. There are disadvantages using periarticular regions compared with the whole hand, however, which include poorer precision and poorer feasibility [33]. Because of skewed data, median values were used instead of mean values, neutralizing the effect of extremes on the BMD results.

The limitations of the present study were that relatively few patients had short disease duration. The effect of medication on the bone was also difficult to evaluate because patients had no standardized treatment but were treated according to clinical judgment. Adjusting for medication use in the multivariate analyses had no significant effect on BMD change either on the DXR-BMD hand or the DXA-BMD hand. A study with a randomized controlled design would give stronger evidence for the effects of medication.

Onepotential limitation using quantitative bone measures as an outcome measure in RA is the influence of normal bone loss, which also takes place in healthy adult subjects. Normal bone loss for DXR-BMD has only been examined in cross-sectional studies reporting an annual rate of bone loss between 0.4% and 0.9% [22,34-36]. For DXA-BMD hip and spine bone loss, using cross-sectional data has been shown to overestimate the rate ofnormal bone loss compared with longitudinal studies [37]. In the multivariate model, however, age was not a significant predictor for hand bone loss over 2 years either for DXR-BMD or for DXA-BMD (data not shown).

## Conclusion

We suggest that hand DXA-BMD can only be used as an outcome measure in RA in the first years of the disease, whereas DXR-BMD may be used as a marker for disease activity and bone loss during the whole disease process, both in early disease as well as prolonged disease. The reason for this discrepancy is not clear and additional studies are warranted to further explore this hypothesis.

## Abbreviations

AOT = antiresorptive osteoporotic treatment; BMD = bone mineral density; DAS 28 = disease activity score including 28 joints; DMARD = disease-modifying antirheumatic drugs; DXA = dual-energy X-ray absorptiometry; DXR = digital X-ray radiogrammetry; IL = interleukin; MCI = metacarpal cortical index; MHAQ = Modified Health Assessment Questionnaire; RA = rheumatoid arthritis; TNF = tumor necrosis factor.

## Competing interests

The authors declare they have no competing interests.

## Authors' contributions

MH analyzed the data, performed the statistical analyses and prepared the manuscript.

TKK and GH designed the study, organized the data collection and contributed substantially to the drafting of the manuscript. All authors read and approved the final manuscript.
